# Sodium Leak Channel in the Nucleus Accumbens Modulates Ethanol-Induced Acute Stimulant Responses and Locomotor Sensitization in Mice: A Brief Research Report

**DOI:** 10.3389/fnins.2021.687470

**Published:** 2021-07-14

**Authors:** Yujie Wu, Donghang Zhang, Jin Liu, Yaoxin Yang, Mengchan Ou, Bin Liu, Cheng Zhou

**Affiliations:** ^1^Laboratory of Anesthesia and Critical Care Medicine, Translational Neuroscience Center, West China Hospital, Sichuan University, Chengdu, China; ^2^Department of Anesthesiology, West China Hospital, Sichuan University, Chengdu, China

**Keywords:** NALCN, nucleus accumbens, ethanol, acute responses, locomotor sensitization

## Abstract

Ethanol can induce acute stimulant responses in animals and human beings. Moreover, repeated exposure to ethanol may produce increased sensitivity to its acute locomotor stimulant actions, a process referred to as locomotor sensitization. The molecular mechanism of the development of acute stimulant responses and locomotor sensitization by ethanol is not fully understood. Sodium leak channel (NALCN) is widely expressed in central nervous system and controls the basal excitability of neurons. The present study aims to determine whether NALCN is implicated in the ethanol-induced acute responses and locomotor sensitization in mice. Here, our results showed that ethanol caused acute stimulant responses in DBA/2 mice. Locomotor sensitization was successfully induced following the sensitization procedure. Accordingly, the expression levels of NALCN mRNA and protein in the nucleus accumbens (NAc) were markedly increased in the sensitization mice compared to the control mice. Knockdown the expression levels of NALCN in the NAc alleviated both the ethanol-induced acute responses and locomotor sensitization. Our findings indicate that upregulation of NALCN expression in the NAc contributes to the ethanol-induced acute stimulant responses and locomotor sensitization in DBA/2 mice.

## Introduction

Ethanol is one of the most commonly abused substances (Abrahao et al., 2017). Although environmental and genetic factors were believed to contribute to the etiology of alcohol use disorders (AUD), the molecular bases of ethanol’s actions was not fully known, including acute stimulant responses and chronic changes in behavior, such as escalated use, compulsive seeking, tolerance, and dependence (Abrahao et al., 2017). Ethanol can induce acute stimulant effects in humans and animals under low to moderate doses (Rodd et al., 2004; Addicott et al., 2007). Moreover, repeated exposure to ethanol will cause locomotor sensitization, which is a defining characteristic of AUD, as displayed by increased sensitivity to the acute locomotor stimulant actions of ethanol after repeated administrations (Robinson and Berridge, 1993). Animal models of locomotor sensitization have been commonly used to assess motivational salience induced by repeated exposure to drugs of abuse, including ethanol (Abrahao et al., 2013; Coelhoso et al., 2013). Currently, the molecular mechanism of the development of both ethanol-induced acute responses and locomotor sensitization remains unclear. Locomotor sensitization is generally believed to be mediated by molecular and morphological changes within reward-related brain nuclei. The nucleus accumbens (NAc) is recognized as the primary striatal subregion that regulates responses to reward (Nestler, 2001; Brady et al., 2003; Kuo et al., 2016). Many studies have highlighted the importance of NAc in regulating the process of drug-induced locomotor sensitization, such as methamphetamine and ethanol (Rose et al., 2013; Su et al., 2019; Ferreira et al., 2021; Porru et al., 2021).

NALCN is a tetrodotoxin (TTX)-resistant and non-selective cation channel that generates a “leak” inward current under physiological conditions (Lu et al., 2007; Ren, 2011). NALCN is widely expressed in the central nervous system and regulates neuronal excitability (Lu et al., 2007). NALCN modulates important functions such as locomotor behaviors (Xie et al., 2013), respiratory rhythm (Lu et al., 2007), and responsiveness to general anesthetics (Jospin et al., 2007; Singaram et al., 2011; Ou et al., 2020; Yang et al., 2020). Previous studies reported that responses to ethanol was changed in NALCN mutations of both animals and humans (Morgan and Sedensky, 1995; Speca et al., 2010; Wetherill et al., 2014; Baronas et al., 2018). The UNC79 heterozygous mice, which is an important auxiliary subunit of NALCN, exhibited acute hypersensitivity to and increased voluntary consumption of ethanol (Speca et al., 2010), suggesting that NALCN may modulate the ethanol actions. However, it is not clear whether NALCN is the key ion channel of NAc to regulate ethanol-induced actions. Here, we put forward a hypothesis that NALCN in NAc may contribute to the ethanol-induced acute stimulant responses and locomotor sensitization.

## Materials and Methods

### Animals

The experimental protocol was approved by the Animal Ethics Committee of West China Hospital of Sichuan University (Chengdu, Sichuan, China) and was conducted in accordance with the Animal Research: Reporting of *In Vivo* Experiments (ARRIVE) guidelines. All performed procedures were approved by the Institutional Animal Care Committee of the Sichuan University. DBA/2 mice (male, ∼8 weeks, 20–25 g) were imported from Jackson Laboratory (United States) and cultivated in Beijing Huafukang Bioscience Company (Beijing, China) over three generations. The mice were maintained under a 12 h (7:00–19:00) light/dark cycle at a constant humidity (45–55%) and temperature (22–24°C) with food and water available *ad libitum*. A maximum of six mice were housed in per standard Plexiglas cage.

### Virus Injection

Adeno-associated virus (AAV) used in this experiment was approved by the Institutional Biosafety Committee of Sichuan University. Mice were anesthetized with pentobarbital sodium (100 mg/kg, i.p.) and fixed in a stereotaxic frame. The skull of mice was drilled and a pipette filled with pAAV2-H1-shRNA-(NALCN)-CAG-eGFP (AAV-NALCN-shRNA) or pAAV2-scrambled-CAG-eGFP (AAV-scrambled-shRNA) virus (2 × 10^13^ TU/ml) was injected bilaterally into the NAc (bregma: -1.3 mm, lateral: ±0.9 mm, depth: -4.7 mm). A total volume of 0.3 μL was injected into each side with the speed of 0.05 μL/min. The sequences of shRNA hairpin used in this study were previously described (NALCN shRNA: AAGATCGCACAGCCTCTTCAT; scrambled: GCTCAGTACGATCATACTCAC) (Shi et al., 2016). Mice were allowed to recover for at least 3 weeks before behavioral experiments.

### Behavioral Tests

Locomotor activity testing was conducted using the mouse autonomic activity tester (TechMan, China). Locomotor activity was detected by interruption of intersecting photocell beams evenly spaced along the walls of the test chamber (300 mm × 120 mm × 100 mm). This equipment was situated in sound-attenuating room equipped with a house light and fan for ventilation and background noise. The locomotor activity testing equipment was interfaced with a Dell computer. Testing continued for 30 min during which time consecutive photocell beam interruptions were translated into number of activities by the computer program. Data were collected in 5-min time intervals. Behavioral tests were performed in the same period from 9:00 am to 12:00 pm.

### Ethanol Administration

Ethanol was diluted with normal saline (0.9% NaCl) to a concentration of 20% (w/v). The procedure of ethanol sensitization used in this study was previously described (Melón and Boehm II, 2011), which is also shown in Table 1. The mice were randomly assigned to the normal saline group (NS), or the ethanol group. The mice were habituated to the intraperitoneal (i.p.) injections and the locomotor activity chambers for 2 days. Then, mice from both groups were intraperitoneally injected with NS to test the baseline locomotor activities (day 0). During the acquisition phase (day 1–day 10), locomotor activities were tested in mice after daily i.p. injection of ethanol (2 g/kg) or equal volume of NS in the ethanol group and/or NS group, respectively. The locomotor activities tested on day 1 after ethanol injection was regarding as the acute responses. On day 10 and day 11, locomotor sensitization was tested by comparation of locomotor activities with day 1 in the ethanol group.

**TABLE 1:** The procedure of ethanol induction.

Groups	Habituation	Baseline	Acute ethanol	Daily treatments (induction)	Sensitization (verification)
Time points	2 days	Day 0	Day 1	Days 2–10	Day 11
NS group	NS	NS	NS	NS	NS
Ethanol group	NS	NS	Ethanol (2 g/kg)	Ethanol (2 g/kg)	Ethanol (2 g/kg)

*NS, normal saline.*

### Real-Time PCR

Total RNA was extracted using the Eastep^®^ Super RNA extraction kit (Promega, Shanghai, China). Reverse transcription was performed with a GoScript^TM^ Reverse Transcription Kit (Promega, Shanghai, China). RT-PCR was performed with the GoTaq^®^ qPCR Master Mix (Promega, Shanghai, China) and specific primers (Sangon Biotech, Shanghai, China) according to the manufacturer’s protocol. The relative fold change of gene expression was calculated with 2^–Δ^ ^Δ^ ^*Ct*^ method with GAPDH as the internal control (Livak and Schmittgen, 2001). The primers used to detect NALCN, UNC79, UNC80 and GAPDH mRNA were as follows:

NALCN forward (5′-GTCCTGACGAATCTCTGTCAGA-3′),

NALCN reverse (5′-CTGAGATGACGCTGATGATGG-3′);

UNC79 forward (5′-CTCCTAGTAGTCTCTGGACCAC-3′),

UNC79 reverse (5′-TGGAAGGTATCTTCAAGAGGCAA-3′);

UNC80 forward (5′-AAGCCTCTTGTGTGTCCTTTG-3′),

UNC80 reverse (5′-CTGTTTGATAGCAGAGTTGCAGT-3′);

GAPDH forward (5′-GACATGCCGCCTGGAGAAAC-3′),

GAPDH reverse (5′-AGCCCAGGATGCCCTTTAGT-3′).

### Immunofluorescence Staining

Mice were anesthetized with pentobarbital sodium (100 mg/kg, i.p.) and were transcardially perfused with ice-cold Ringer’s solution followed by 4% paraformaldehyde. The brain was removed and stored in a 4% paraformaldehyde solution overnight, followed by incubation in 30% sucrose for 1 day. Transverse sections (12 μm) were cut using a freezing microtome (CM1850; Leica, Buffalo Grove, IL, United States). Sections were incubated at 4°C overnight with the following primary antibodies: NALCN (1:400, rabbit, Alomone Labs, Israel, Cat#: ASC022), NeuN (1:400, mouse, Millipore, United States, MAB377), and c-Fos (1:5000, rabbit, Millipore, United States, ABE457). Then, sections were incubated with secondary antibodies for 2 h: Alexa Fluor 488 goat anti-mouse IgG (1:400, Millipore, United States), or Fluor 647 goat anti-rabbit IgG (1:400, Millipore, United States). Fluorescent images were acquired using Zeiss AxioImager Z.2 (Guangzhou, Guangdong, China).

### Western Blot

The NAc was homogenized in radioimmunoprecipitation assay lysis buffer (Beyotime, China) containing protease inhibitors. The protein concentration was quantified using a BCA Protein Assay Kit (Beyotime, China) according to the instructions. Ten micrograms of protein were separated by 10% polyacrylamide gel electrophoresis and then transferred to polyvinylidene difluoride membranes (Bio-Rad Laboratories, United States). To reduce non-specific binding, the membranes were incubated with Tris–buffered saline/Tween-20 containing 5% non-fat milk at room temperature for 2 h. Subsequently, the membranes were incubated with primary antibodies against NALCN (1:500, rabbit, ASC-022, Alomone Labs, Israel), β-actin (1:1000, rabbit, A2066, Sigma-Aldrich, United States) and GAPDH (1:1000, rabbit, D16H11, Cell Signaling, United States) at 4°C overnight and then with a peroxidase-conjugated goat anti-rabbit IgG secondary antibody (1:5000, Proteintech, China) for 2 h. Immunoreactivity was visualized with Clarity Western ECL Substrate (Bio-Rad Laboratories) and Amersham Imager 600 (General Electric, United States). ImageJ software (National Institutes of Health, United States) was used for densitometry measurements.

### Statistical Analysis

Data were present as means ± SD and statistical analyses were performed by GraphPad Prism software 8.0 or SPSS software 18.0. Parametric distribution data or non-parametric distribution data between two groups were compared by paired/unpaired Student’s *t*-tests or Mann-Whitney *U* test, respectively. For analysis of multiple groups at single time points, one-way ANOVA was used, followed by Tukey-Kramer *post hoc* multiple comparisons test. Behavioral tests (multiple groups at multiple time points) were analyzed using a two-way analysis of variance (ANOVA) with repeated measures followed by a Bonferroni *post hoc* test. *P* < 0.05 was considered statistically significant.

## Results

### Expression Level of NALCN mRNA in NAc Increases After Repeated Exposure to Ethanol in Mice

**FIGURE 1:**
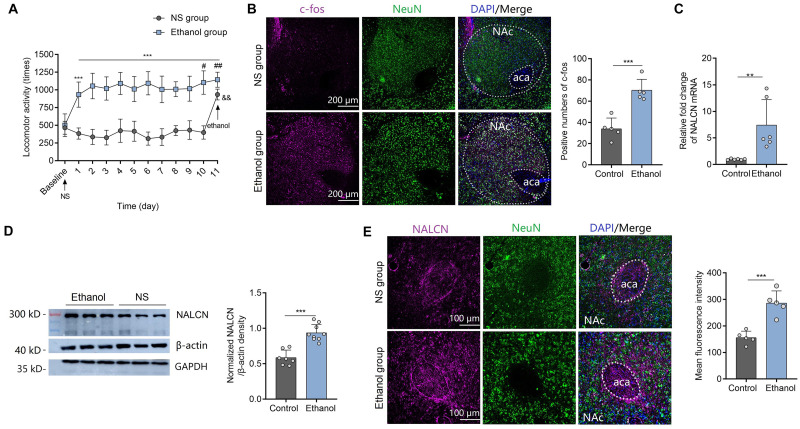
Expression of NALCN increases after ethanol-induced locomotor sensitization. **(A)** Ethanol injection induced acute stimulant locomotion compared to NS. Repeated treatment with ethanol induced locomotor sensitization (*n* = 10; *t* = 5.71, *df* = 18, ***P < 0.001 compared with baseline; *F*_sensitization_(1,9) = 740.45, ***P < 0.001 compared with NS group; *t* = 2.22, *df* = 18, ^#^*P* < 0.05 compared with day 1 in the ethanol group; *t* = 3.20, *df* = 18, ^##^*P* < 0.01 compared with day 1 in the ethanol group; *t* = 3.00, *df* = 18, ^&&^*P* < 0.01 compared with the ethanol group). **(B)** Representative images of c-Fos staining (left) showed that c-Fos expression was greater in the NAc of sensitization mice after ethanol injection, compared to the control mice (right, *n* = 5, *t* = 5.62, *df* = 8, ***P < 0.001). **(C)** Expression of NALCN mRNA in the NAc was significantly increased after repeated exposure to ethanol (*n* = 6, *t* = 3.32, *df=10*, **P < 0.01). **(D)** Expression of NALCN protein in the NAc was significantly increased after repeated exposure to ethanol (*n* = 7−9, *t* = 6.19, *df=14*, ***P < 0.001). **(E)** Representative images of NALCN staining (left) showed that NALCN expression was greater in the NAc of sensitization mice after ethanol injection, compared to the control mice (right, *n* = 5, *t* = 5.61, *df* = 8, ***P < 0.001). Data are present as mean ± SD. Panel **(A)** was compared by two-way ANOVA; **(B)** right, **(C,D)** right, and **(E)** right were compared by unpaired two-tailed student’s *t*-test. The results contained three replicates across cohorts of mice.

On day 0 (baseline), there was no difference in basal locomotor in the mice of ethanol group and NS group after i.p. injection of NS (*t* = 0.19, *df* = 18, *P* = 0.8517) (Figure 1A). On day 1, ethanol injection induced significant acute stimulant responses as evidenced by more locomotor activities compared to NS control (Figure 1A). During the acquisition phase, all ethanol-treated animals presented increased locomotion compared to NS control from day 1 to 10 [factor time: *F*(9,81) = 1.39, *P* = 0.21; factor sensitization: *F*(1,9) = 740.45, *P* < 0.001; interaction: *F*(9,81) = 1.33, *P* = 0.23] (Figure 1A). Locomotor activities on day 10 (*t* = 2.22, *df* = 18, *P* < 0.05) and day 11 (*t* = 3.20, *df* = 18, *P* < 0.01) after ethanol injection were significantly increased compared to that on day 1 in the ethanol group (Figure 1A), indicating that repeated ethanol injections induced locomotor sensitization. On day 11, ethanol was injected to the mice of both NS group and ethanol group. More locomotor activities were observed in the ethanol group than the NS group (*t* = 3.00, *df* = 18, *P* < 0.01) (Figure 1A), which further confirmed the successful establishment of locomotor sensitization. In the ethanol group, c-Fos expression in the NAc was greater than that of control mice at 1.5 h after ethanol injection on day 11 (*t* = 5.62, *df* = 8, *P* < 0.001) (Figure 1B), suggesting that neurons in NAc were activated by ethanol. Next, we determined the NALCN expression level in the mice with locomotor sensitization. Expression of NALCN mRNA (*t* = 3.32, *df=10*, *P* < 0.01) (Figure 1C) and protein (Western Blot with β-action as the internal control: *t* = 6.19, *df=14*, *P* < 0.001; Immunofluorescence results: *t* = 5.61, *df* = 8, *P* < 0.001) (Figures 1D,E) in the NAc was significantly increased after repeated exposure to ethanol, compared to control mice. As one study demonstrated that β-action is a poor loading control for western blot quantification despite its widespread use (Dittmer and Dittmer, 2006), we reproduced the results with another known internal control-GAPDH (Supplementary Figure 1A). In addition, we found the trend between locomotor activities and expression levels of NALCN was positive, but not significantly linear correlated (For mRNA: *r* = 0.325, *P* = 0.53. For protein: *r* = 0.495, *P* = 0.32) (Supplementary Figure 1B).

### Knockdown the Expression Level of NALCN mRNA in NAc Alleviates Ethanol-Induced Acute Stimulant Responses and Locomotor Sensitization in Mice

**FIGURE 2:**
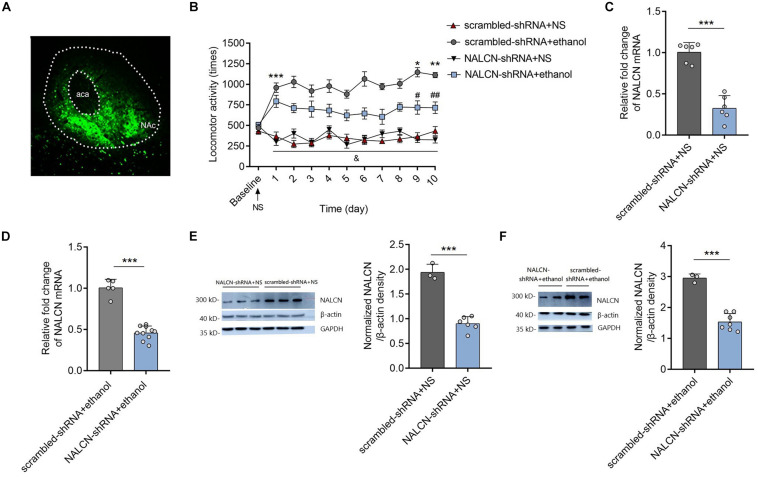
Knockdown the NALCN expression alleviated the ethanol induced acute stimulant responses and locomotor sensitization. **(A)** Representative images of AAV-GFP expression in NAc 4 weeks after injection. **(B)** Knockdown the NALCN expression in NAc by AAV-NALCN-shRNA significantly inhibited the ethanol induced acute stimulant responses and locomotor sensitization [*n* = 10, *t* = 6.69, *df* = 18, ***P < 0.001 compared with baseline; *t* = 2.31, *df* = 18, *P < 0.05 compared with day 1 in the AAV-scrambled-shRNA + ethanol group; *t* = 2.31, *df* = 18, **P < 0.05 compared with day 1 in the AAV-scrambled-shRNA + ethanol group; *t* = 2.45, *df* = 18, ^#^*P* < 0.05 compared with the AAV-scrambled-shRNA + NS group; *t* = 2.33, *df* = 18, ^##^*P* < 0.05 compared with the AAV-scrambled-shRNA + NS group; ^&^*P* < 0.05 for AAV-NALCN-shRNA+NS group (*F*_interaction_(9,81) = 2.47) or AAV-scrambled-shRNA+NS group (*F*_interaction_(9,81) = 2.20) compared with the AAV-scrambled-shRNA+ethanol group]. **(C,D)** Expression levels of NALCN mRNA was significantly decreased in NAc by AAV-NALCN-shRNA [**(C)**: *n* = 6; *t* = 8.63, *df* = 10, ***P < 0.001; **(D)**: *n* = 5–11; *t* = 11.09, *df* = 13, ***P < 0.001]. **(E,F)** Expression levels of NALCN protein was significantly decreased in NAc by AAV-NALCN-shRNA [*n* = 3–7, **(E)**: *t* = 9.77, *df* = 7, ***P < 0.001; **(F)**: *t* = 8.39, *df* = 8, ***P < 0.001]. Data are present as mean ± SD. Panel **(A)** was compared by two-way ANOVA; **(C,D)** right and **(E)** right were compared by unpaired two-tailed student’s *t*-test. The results contained three replicates across cohorts of mice.

To test the causality between NALCN expression and the development of ethanol-induced acute stimulant responses and locomotor sensitization, AAV-NALCN-shRNA or AAV-scrambled-shRNA virus was injected into the NAc of mice. GFP-positive fluorescence was detected in the NAc 4 weeks after AAV injection (Figure 2A). We found that knockdown the NALCN expression in NAc significantly inhibited the acute stimulant actions induced by ethanol (Figure 2B). Repeated ethanol-induced locomotor sensitization was alleviated by the AAV-NALCN-shRNA [factor time: *F*(9,81) = 1.28, *P* = 0.236; factor shRNA: *F*(1,9) = 32.54, *P* < 0.001; interaction: *F*(9,81) = 1.91, *P* < 0.05] (Figure 2B). The results also showed that knockdown of NALCN in NAc produced no effects on locomotor activities in the mice that treated with NS over time [factor time: *F*(9,81) = 1.03, *P* = 0.43; factor shRNA: *F*(1,9) = 0.64, *P* = 0.443; interaction: *F*(9,81) = 1.78, *P* = 0.086] (Figure 2B). Accordingly, NALCN mRNA (NS treated: *t* = 8.63, *df* = 10, *P* < 0.001; ethanol treated: *t* = 11.09, *df* = 13, *P* < 0.001) (Figures 2C,D) and protein (with β-action as the internal control, NS treated: *t* = 9.77, *df* = 7, *P* < 0.001; ethanol treated: *t* = 8.39, *df* = 8, *P* < 0.001) (Figures 2E,F) levels was significantly decreased in NAc by AAV-NALCN-shRNA compared to AAV-scrambled-shRNA. The western blot results were consistent when another internal control-GAPDH was used (Supplementary Figure 1C). These results revealed that NALCN in the NAc modulates the acute stimulant actions of ethanol and upregulation of NALCN expression in the NAc contributes to the ethanol-induced locomotor sensitization in DBA/2 mice.

## Discussion

The present study demonstrates that NALCN in NAc can modulate the ethanol-induced acute stimulant responses and chronic locomotor sensitization in DBA/2 mice. Repeated ethanol exposure can induce locomotor sensitization, accompanied by an increased neuronal activation in the NAc (Ferreira et al., 2021). Consistently, we also observed that the expression of c-Fos protein is greater in NAc after repeated exposure to ethanol. NALCN is widely expressed in the central nervous system and regulates neuronal excitability (Lu et al., 2007). Several studies indicated that NALCN implicated in the ethanol responses of both animals and humans being (Speca et al., 2010; Cochet-Bissuel et al., 2014; Wetherill et al., 2014; Baronas et al., 2018). One single nucleotide polymorphism (SNP) in NALCN has been found to strongly associate with the high risk of alcohol dependence (Wetherill et al., 2014). We hypothesize that NALCN might be a key ion channel in NAc that mediates the actions of ethanol. Here we showed that NALCN expression was upregulated in the NAc of mice along with locomotor sensitization. Knockdown the NALCN expression in the NAc significantly alleviated the locomotor sensitization in mice *in vivo*. In addition, the acute stimulant responses induced by ethanol were markedly inhibited after NALCN knockdown. Therefore, NALCN is a potential target for ethanol to induce acute stimulant responses and chronic locomotor sensitization.

UNC79 and UNC80 are two important auxiliary subunits of NALCN (Ren, 2011). Mutation in one of these three genes can affect the protein levels and/or localization of the others (Ren, 2011). Previous study reported that acute hypersensitivity to ethanol was observed in UNC79, UNC80, and NCA-1/NCA-2 (orthologs of NALCN) double mutants of *Caenorhabditis elegans* and UNC79 heterozygous mice (Speca et al., 2010), suggesting a conserved pathway that might influence alcohol-related behaviors. Moreover, acute locomotor activation in response to ethanol was not detected in UNC79 heterozygous mice (Speca et al., 2010). Consistent with this, our results revealed that knockdown NALCN in NAc can prevent the acute locomotor activity in DBA/2 mice. These results suggest that NALCN may be a potential molecular target of ethanol-induced acute stimulant actions. Interestingly, UNC79 heterozygous mice exhibited increased voluntary consumption of ethanol compared to wild-type mice (Speca et al., 2010). We also found that mRNA levels of UNC79, but not UNC80 in the NAc were increased after repeated exposure to ethanol (Supplementary Figure 1D). It is possible that ethanol may directly activate NALCN/UNC79 to release dopamine which may cause a higher ethanol preference in mice. However, because the UNC79 protein is widely expressed throughout the central nervous system, it is unlikely to test the contribution of specific sites, such as NAc, in the ethanol actions. Nonetheless, it is believed that normal NALCN expression or function is required for ethanol responses. Exploring of these hypotheses in future may provide insights into the mechanism of ethanol actions on the nervous system and specifically into the mechanisms of reward-related behaviors.

Some limitations exist in our study. Firstly, whether NALCN in other reward-related brain nuclei, such as VTA, also contributes to ethanol-induced responses was unknown. Secondly, whether knockdown of NALCN expression in NAc affects normal physiological functions remains unclear although no detrimental phenotypes were observed in our study. It is also should be concerned that we did not test whether upregulation of NALCN expression in the NAc contributes to the ethanol-induced acute stimulant responses and locomotor sensitization in female DBA/2 mice.

In conclusion, we found that NALCN is an important ion channel for ethanol-induced acute stimulant responses and locomotor sensitization. This study may provide novel insights into the mechanism of alcohol actions, and studies of NALCN gene in humans may lead to a better understanding of alcoholism and its treatment.

## Data Availability Statement

The original contributions presented in the study are included in the article/Supplementary Material, further inquiries can be directed to the corresponding author.

## Ethics Statement

The experimental protocol was approved by the Animal Ethics Committee of West China Hospital of Sichuan University (Chengdu, Sichuan, China) and was conducted in accordance with the Animal Research: Reporting of *In Vivo* Experiments (ARRIVE) guidelines.

## Author Contributions

All authors listed have made a substantial, direct and intellectual contribution to the work, and approved it for publication.

## Conflict of Interest

The authors declare that the research was conducted in the absence of any commercial or financial relationships that could be construed as a potential conflict of interest.

## Supplementary Material

Supplementary Figure 1 **(A)** Expression of NALCN protein (normalized by GAPDH) in the NAc was significantly increased after repeated exposure to ethanol (*n* = 4–7, *t* = 4.15, *df* = 9, ^∗∗^*P* < 0.01). **(B)** Locomotor activities were positively correlated with the expression levels of NALCN (For mRNA: *r* = 0.325, *P* = 0.53; For protein: *r* = 0.495, *P* = 0.32). **(C)** Expression levels of NALCN protein (normalized by GAPDH) was significantly decreased in NAc by AAV-NALCN-shRNA (*n* = 3–7, right: *t* = 9.47, *df* = 7, ^∗∗∗^*P* < 0.001; left: *t* = 3.85, *df* = 8, ^∗∗^*P* < 0.01). **(D)** Expression levels of UNC79 mRNA in the NAc was significantly increased after repeated exposure to ethanol (*n* = 4–6, *t* = 3.02, *df* = 8, ^∗^*P* < 0.05). No significant difference was found in the expression of UNC80 mRNA (*n* = 4–6, *t* = 1.27, *df* = 8, *P* = 0.24). Data are present as mean ± SD. Panels **(A,C,D)** were compared by unpaired two-tailed student’s *t*-test. Panel **(B)** was analyzed by linear regression.
