# Supplementary figures and images for: Sodium Leak Channel in the Nucleus Accumbens Modulates Ethanol-Induced Acute Stimulant Responses and Locomotor Sensitization in Mice: A Brief Research Report
Source: Front Neurosci. 2021 Jul 14;15:687470. doi: 10.3389/fnins.2021.687470 (PMC8316816; doi:10.3389/fnins.2021.687470)

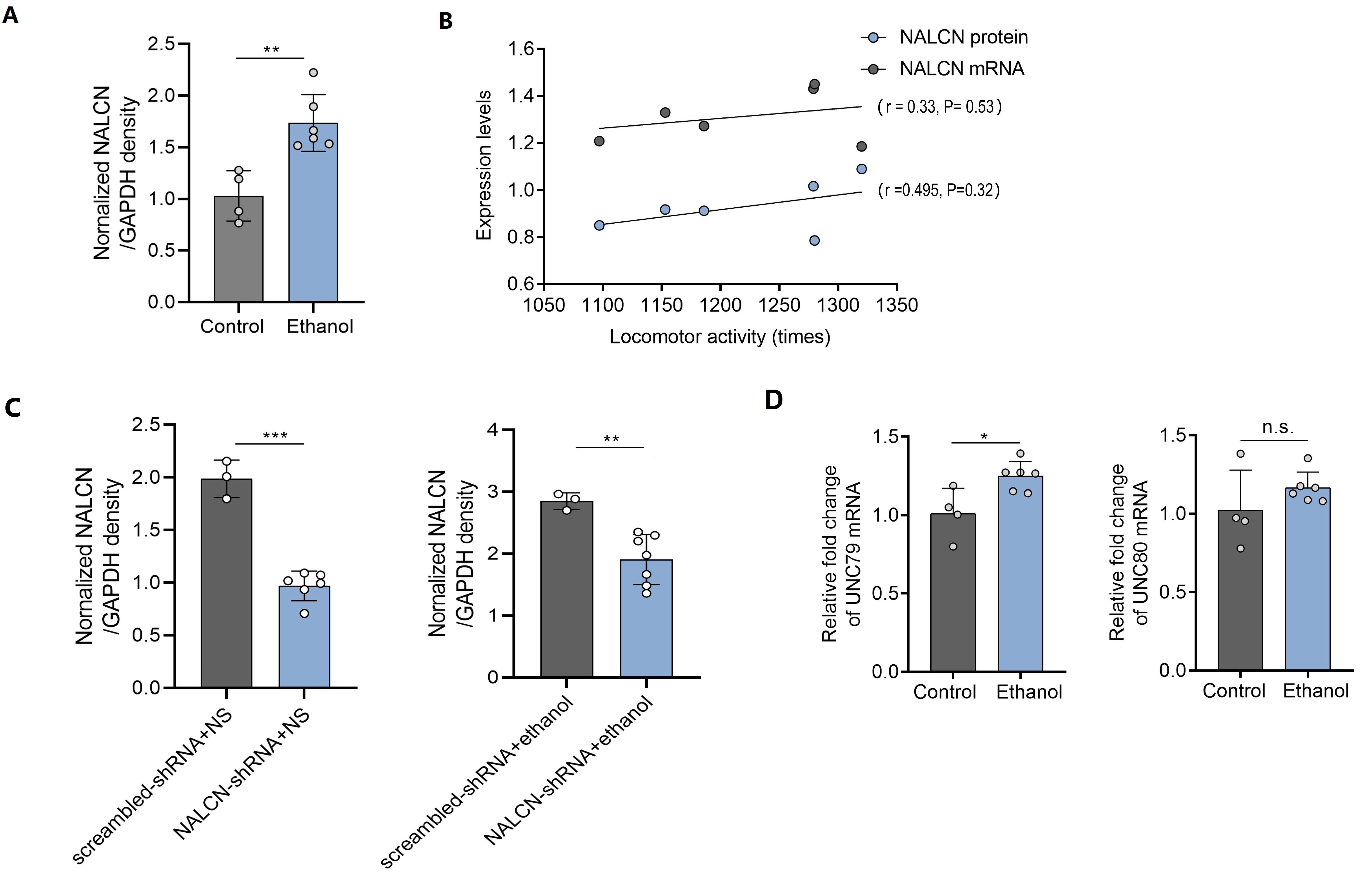

Supplement: Supplementary Figure 1 — (A) Expression of NALCN protein (normalized by GAPDH) in the NAc was significantly increased after repeated exposure to ethanol (n = 4–7, t = 4.15, df = 9, ∗∗P < 0.01). (B) Locomotor activities were positively correlated with the expression levels of NALCN (For mRNA: r = 0.325, P = 0.53; For protein: r = 0.495, P = 0.32). (C) Expression levels of NALCN protein (normalized by GAPDH) was significantly decreased in NAc by AAV-NALCN-shRNA (n = 3–7, right: t = 9.47, df = 7, ∗∗∗P < 0.001; left: t = 3.85, df = 8, ∗∗P < 0.01). (D) Expression levels of UNC79 mRNA in the NAc was significantly increased after repeated exposure to ethanol (n = 4–6, t = 3.02, df = 8, ∗P < 0.05). No significant difference was found in the expression of UNC80 mRNA (n = 4–6, t = 1.27, df = 8, P = 0.24). Data are present as mean ± SD. Panels (A,C,D) were compared by unpaired two-tailed student’s t-test. Panel (B) was analyzed by linear regression. [file Image_1.JPEG]
